# Synergistic Neuroprotective and Immunomodulatory Effects of Cocoa Seed Husk and Guarana Extract: A Nutraceutical Approach for Parkinson’s Disease Management

**DOI:** 10.3390/antiox14030348

**Published:** 2025-03-15

**Authors:** Vitória Farina Azzolin, Verônica Farina Azzolin, Euler Esteves Ribeiro, Juliane Santiago Sasso, Douglas Reis Siqueira, Nathalia Cardoso de Afonso Bonotto, Bárbara Osmarin Turra, Marco Aurélio Echart Montano, Ednea Aguiar Maia Ribeiro, Raquel de Souza Praia, Maria Fernanda Mânica-Cattani, Cristina Maranghello, Railla da Silva Maia, Erickson Oliveira dos Santos, Pedro Luis Sosa Gonzalez, Cleideane Cunha Costa, Vanusa Nascimento, Fernanda Barbisan, Ivana Beatrice Mânica da Cruz

**Affiliations:** 1 Research, Teaching and Technological Development Center, Fundação Universidade Aberta da Terceira Idade, Manaus 69029-040, Brazil; vitoriaazzolin@hotmail.com (V.F.A.); veronica.azzolin@funati.am.gov.br (V.F.A.); euler.ribeiro@funati.am.gov.br (E.E.R.); sassojuliane@gmail.com (J.S.S.); dougreisbio@gmail.com (D.R.S.); nathalia.bonotto@acad.ufsm.br (N.C.d.A.B.); babi_turra@hotmail.com (B.O.T.); echartmontano@gmail.com (M.A.E.M.); edneamaiaribeiro@gmail.com (E.A.M.R.); raquelsouzapraia@gmail.com (R.d.S.P.); fernanda18cattani@gmail.com (M.F.M.-C.); cristinamaranghello@gmail.com (C.M.); raillamaia@hotmail.com (R.d.S.M.); vanusa.nascimento@funati.am.gov.br (V.N.); fernandabarbisan@gmail.com (F.B.); 2 Graduate Program in Biotechnology, Universidade Federal do Amazonas, Manaus 69080-900, Brazil; 3 Postgraduate Program in Gerontology, Universidade Federal de Santa Maria, Santa Maria 97105-900, Brazil; 4 Biogenomics Laboratory, Pathology Department, Universidade Federal de Santa Maria, Santa Maria 97105-900, Brazil; 5 Centro Universitário da Serra Gaúcha, Caxias do Sul 95020-472, Brazil; 6 Postgraduate Program in Aging Sciences—Universidade São Judas Tadeu, São Paulo 03166-000, Brazil; 7 Samsung Research & Development Institute Brazil, Manaus 69055-035, Brazil; erickson.o@samsung.com (E.O.d.S.); pedro.sosa@samsung.com (P.L.S.G.); cleide.c@samsung.com (C.C.C.)

**Keywords:** functional foods, *Theobroma cacao*, *Paullinia cupana*, flavonoids

## Abstract

Background: Parkinson’s disease (PD) is a progressive neurodegenerative disorder linked to oxidative stress, mitochondrial dysfunction, and neuroinflammation. This study evaluates the neurofunctional and immunomodulatory effects of an aqueous extract combining cocoa seed husk and guarana powder (GuaCa). Eighteen extracts were characterized by flavonoid and polyphenol content, antioxidant activity, and genoprotective potential. The HCE3 extract, rich in catechins, quercetin, and epigallocatechin gallate, was selected for further analysis in three models: *Eisenia fetida* earthworms, SH-SY5Y neuron-like cells, and peripheral blood mononuclear cells (PBMCs) from PD patients. Results: The extracts showed antioxidant and genoprotective activity and contained flavonoid. No significant toxicity was observed in *Eisenia fetida*. In SH-SY5Y cells, GuaCa increased cell viability and brain-derived neurotrophic factor (BDNF) levels and reduced mitochondrial damage by lowering extracellular NDUSF7 (subunit of the NADH dehydrogenase (ubiquinone) complex) levels. In dPD-PBMCs cultures, GuaCa reduced pro-inflammatory cytokine IL-6 levels, indicating immunomodulatory effects. Conclusion: GuaCa shows promise as a nutraceutical for managing neuroinflammation and mitochondrial dysfunction in PD. Further clinical studies are needed to confirm GuaCa extract efficacy and potential for neuroprotective dietary strategies.

## 1. Introduction

The prevalence of Parkinson’s disease (PD) is significantly increasing, affecting about 1–2% of the world’s older adult population [1]. Although complex, the pathogenesis of this still incurable disease is directly linked to the degeneration of dopaminergic neurons in the brain’s substantia nigra. This neurodegeneration appears to be triggered by states of oxidative stress, which in turn lead to mitochondrial dysfunction. This mainly occurs in Complex I of oxidative phosphorylation, in the oxidation of strategic molecules neurotransmission as dopamine, and the conformational alteration in a large number of proteins [2]. These alterations contribute to the production and deposition of cytosolic inclusion bodies primarily composed of the protein α-synuclein (Lewy bodies) and black pigments known as neuromelanin [3].

The mitochondrial dysfunction and accumulation of these modified molecules have been directly associated with neuronal death in several ways, such as apoptosis and pyroptosis [3]. Both damaged mitochondria and other residues are expelled from the interior of cells into the extracellular environment, probably contributing to Damage-associated Molecular Patterns (DAMPs) leading to states of neuroinflammation and systemic inflammation present in PD [4]. From there, these circuits of oxidative stress, mitochondrial dysfunction, and molecule damage reinforce each other, leading to the onset of PD’s clinical symptoms and progression.

Within this context, a strategy to mitigate the impact of oxidative-inflammatory states and mitochondrial dysfunction in PD could involve ingesting foods or supplements rich in antioxidant and anti-inflammatory bioactive molecules. Evidence from pre-clinical and clinical studies indicates that the consumption of various antioxidant molecules, especially those in the flavonoid category, can benefit PD by reducing oxidative and inflammatory states perpetuated during the disease [5]. In this context, developing nutritional supplements rich in flavonoids and proteins could benefit these patients [6].

A potential source of these molecules involves agro-industrial byproducts generated in the processing of fruits. These residues possess a significant volume of residues, generally constituting 65% to 70% of the total fruit mass [7]. This is the case of some Amazonian diet fruits, such as cocoa shell bean (CBS) (*Theobroma cacao* L.), which contains polyphenols, methylxanthines (especially theobromine), carbohydrates (fibers), and proteins in its chemical matrix [8]. Thus, it is possible that a combined extract of cocoa seed husk and guarana seed powder could produce a formulate rich in flavonoids with relevant functional properties to PD, including improvement of neurofunctional states, reduction in mitochondrial damage, and immunomodulation of pro-inflammatory markers.

Aqueous extracts combining CBS and guarana powder rich in flavonoids (GuaCa) were produced and chemically characterized to test this assumption. A Guaca extract of reference was chosen to evaluate its potential toxicity in an in vivo red Californian earthworm (*Eisenia fetida*) experimental model. Potential neurofunctional and immunomodulatory GuaCa’s effect was also tested in SH-SY5Y neuron-like cultures and peripheral blood mononuclear cells (PBMCs) cultures obtained from volunteers diagnosed with PD. These experimental models have been previously used in studies involving Amazonian food supplements, such as guaraná and açai [9,10,11].

## 2. Materials and Methods

### 2.1. Experimental Design

In order to obtain an extract from CBS with a higher flavonoid content—molecules that are potentially relevant in Parkinson’s disease [5]—different extracts were produced by varying pH and temperature. The chemical characterization of total polyphenols, flavonoids, catechins, as well as the antioxidant and genoprotective capacity of all extracts, was quantified. A representative GuaCa extract, positioned near the 75th percentile of flavonoid concentration, was selected for complementary analyses. The extract with the highest flavonoid concentration was not chosen because, in the production of new extracts under the same experimental conditions, relevant variations in these molecules could occur due to differences in raw material origin, cultivation conditions, or plant variety. Additionally, very high flavonoid levels could be accompanied by elevated concentrations of other micronutrients, which might lead to undesirable effects, such as Zn [12] and Se [13].

Ultra-Performance Liquid Chromatograph (UPLC) coupled with mass spectrometry (MS) (UPLC-QToF-MS, Waters Co., Milford, MA, USA), was used to identify the principal peaks of molecules present in the selected GuaCa extract, which predominantly comprised flavonoids, xanthines, and proteins, including caffeine and catechin. According to the literature, caffeine from guarana extract can present potential beneficial antioxidant and antifatigue effects in PD [14]. The potential toxicity of GuaCa at different concentrations was tested using the Red Californian earthworm (*E. fetida)* as an experimental model. This model is commonly used in ecotoxicity studies and research involving extracts, nutritional formulations, pharmaceuticals, and pollutants [15,16].

The GuaCa concentrations that did not trigger higher earthworm mortality were used to evaluate GuaCa’s in vitro neurofunctional effects assessed in the SH-SY5Y (CRL-2266™, American Type Culture Collection (ATCC, Manassas, VA, USA) human neuron-like cell line. The viability of cells supplemented with GuaCa was determined using the MTT assay. A concentration of GuaCa that caused a potential therapeutic effect, observed through a significant increase in neural viability, was utilized in the subsequent tests. This same concentration was also used in in vitro assays with PBMCs from volunteers diagnosed with PD. Neurofunctional indicators of GuaCa were investigated through the analysis of four biochemical markers.

The first involved quantifying the DNA damage rate, which is elevated in PD, determined by the levels of 8-Hydroxydeoxyguanosine [17]. Although DNA damage is particularly elevated in the substantia nigra of the hypothalamus, patients with Parkinson’s disease (PD) may also exhibit DNA damage in PBMCs [18]. The second involved assessing the rate of mitochondrial damage by quantifying the NDUFS7 protein of Mitochondrial Complex I in the extracellular medium [2]. The third evaluated the effect of GuaCa supplementation on the modulation of brain-derived neurotrophic factor (BDNF) and its tropomyosin receptor kinase type B (TrkB), which are directly involved in the neuropathology of PD. The BDNF promotes the survival of dopaminergic neurons in the substantia nigra and increases the functional activity of striatal neurons. Therefore, BDNF deficiency increases in PD and is associated with disease severity and long-term complications [19]. For this reason, inducing an increase in BDNF levels could be a desirable functional property.

As growing evidence shows the involvement of cellular senescence in the pathogenesis of neurodegenerative diseases, including PD, it was also evaluated whether the GuaCa extract could differentially modulate the enzyme beta-galactosidase (β-Gal) [20].

An additional in vitro protocol also evaluated whether supplementation of the culture medium with GuaCa could exhibit any immunomodulatory properties in peripheral blood mononuclear cells (PBMCs) obtained from volunteers diagnosed with PD. In this protocol, similar to [21], the GuaCa effects on cellular proliferation and the pro-inflammatory cytokine interleukin 6 (IL-6) levels were determined. Additionally, alterations in the cytomorphological patterns of cell size and extracellular residual debris that can indicate PBMCs’ inflammatory activation or degenerative states were evaluated.

### 2.2. GuaCa Extract Preparation

The GuaCa aqueous extracts were produced with variations in the proportion of CBS and roasted ground guarana seeds, as well as adjustments in temperature and pH. Hot extracts were identified with the abbreviation HE, cold extracts with the abbreviation CE, and extracts processed at both temperatures with the abbreviation HCE. The following steps were carried out for the preparation of GuaCa extracts.

Step 1—The two main raw materials were combined to produce a mixture, with cocoa bean shell as the major component and roasted, ground guarana seed powder as the minor component. The proportions ranged from 1:0.5 to 1:10 but were not limited to these ratios. The pH, temperature, and extraction time of each extract produced are summarized in Table 1.

Step 2—The temperature was adjusted to enhance the extraction of flavonoids (primarily at lower temperatures), while also ensuring the extraction of other relevant bioactive molecules, such as caffeine (primarily at higher temperatures). The mixture with water, under agitation, was subjected to an initial temperature of 85 °C for HE extracts. This temperature was chosen because many polyphenols, including flavonoids, can be thermosensitive and degrade at higher temperatures [22]. Cold extracts (CE) were obtained at −4 °C. Alternatively, a combination of both temperature ranges (HCE) was used. The extraction at these temperatures was maintained for 4 h.

Step 3—The pH was adjusted to either acidic or neutral. In general, acidification can enhance the efficiency of certain bioactive molecules, such as polyphenols [23]. Three pH measurements were performed: First pH measurement—taken immediately after mixing the raw material with distilled water. Second pH measurement—taken after 4 h of extraction at either hot or cold temperatures. Third pH measurement—taken after 24 h of extraction, during which the extracts remained at room temperature. The first pH was lowered using citric acid (pH < 5) or neutralized using NaOH (pH = 7.0 ± 2.0). After extraction at hot, cold, or combined hot/cold conditions, the pH was measured again and adjusted accordingly. The third pH assessment, conducted after 24 h, confirmed that the extracts maintained their acidic or neutral pH.

Step 4—Filtration and drying: After the extraction period, the extracts were filtered using Whatman filter paper (Sigma- Aldrich, Saint Louis, MO, USA) and transformed into a powder by lyophilization, using a freezing process at −45 °C, followed by freeze-drying for 24 h under a pressure of 0.036 mbar and a condenser temperature of −104.7 °C.

Step 5—Dilution of the lyophilized powder for bioactive component quantification: The lyophilized extract powder was diluted for the quantification of its bioactive components using spectrophotometry (microplate reader (DR-200BS-NM-BI, Kasuaki, Republic of Korea) and (UPLC-QToF-MS, Waters Co., Milford, MA, USA), proximate analysis, and for conducting in vitro and in vivo experimental protocols. Since the extraction process resulted in particles that do not readily dissolve in solutions at room or cold temperatures, the GuaCa extract powder was redissolved in phosphate buffer and subjected to agitation at 85 °C for 10 min, followed by an additional filtration step.

### 2.3. Materials and Reagents

The CBS was commercially obtained from Jupará Chocolates Artesanais (Salvador, Bahia, Brazil), and the guarana powder was obtained from a Brazilian government-owned company—Embrapa Amazonia Oriental (Maués, Amazonas, Brazil). All chemicals and solvents utilized in this research were sourced from Sigma-Aldrich (Saint Louis, MO, USA), including Histopaque^®^-1077, which isolated PBMCs and protocols involving biochemical reagents. Materials for cell culture experiments were obtained from Vitrocell-Embriolife (Campinas, São Paulo, Brazil). The molecular biology reagents were supplied by Qiagen (Hilden, North Rhine-Westphalia, Germany), Invitrogen (Carlsbad, CA, USA), and Bio-Rad Laboratories (Hercules, CA, USA). DNA calf thymus (dsDNA) was purchased from Invitrogen (Eugene, OR, USA). Enzyme-linked immunosorbent assay (ELISA) kits containing inflammatory markers were purchased from Elabscience (Houston, TX, USA).

### 2.4. Spectophotometric Quantification of Main Chemical Groups

In all protocols, the quantification of polyphenols, flavonoids, and catechins was performed by spectrophotometry assays, and the average of the triplicates was calculated for statistical analysis. The total phenolic content was quantified using Folin–Ciocalteu’s method [24] with a calibration curve prepared from standard gallic acid solutions. The mg GAE/mL calibration curve equation was y = 0.0689x − 0.0482, and r^2^ = 0.9766 was obtained from the following concentrations: 5, 10, 15, 20, 25, 30, 35, 40, 45 mg/mL. The extract samples were measured at wavelengths ranging from 760 to 765 nm. The aluminum chloride colorimetric method was used to determine flavonoids [25]. The data were expressed in µg/mL quercetin equivalents, calculated from a standard curve (y = 0.0098x + 0.0328; r^2^ = 0.9862) calculated from the follow concentrations: 0.2, 0.4, 0.6, 0.8, 1.0 and 1.2 mg/mL.

The vanillin-HCl method was employed to quantify catechins and other condensed tannins based on the reaction between vanillin and the flavan-3-ol groups of catechin, forming a colored complex measurable at 500 nm [26]. The catechin content was expressed in µg/mL. The calibration curve for catechin was obtained using the equation y = 0.0172x + 0.0051, r^2^ = 0.985, calculated from the following concentrations: 0.5, 1.0, 1.5, 2.0, 2.5, 3.0, and 3.5 mg/mL.

### 2.5. Assays of DPPH Antioxidant Capacity and Genomodifier Capacity Assays

The DPPH method (2,2-diphenyl-1-picrylhydrazyl stable free radical) was used to measure the antioxidant capacity of the lyophilized extracts, as previously described [27]. This assay is based on measuring the antioxidant capacity of substances to scavenge the stable DPPH· radical, which can be assessed by monitoring the decrease in its absorbance via spectrophotometry measured at 517 nm. The percentage of unreacted DPPH· represents the antioxidant capacity percentage (%AA) and is proportional to the concentration of the antioxidant substance. The reaction mixture was read in microplates of 96 wells at an absorbance of 520 nm. Three calibration curves were prepared using ascorbic acid (AA, y = 6.6916x + 32.15y = 6.6916x + 32.15y = 6.6916x + 32.15, r^2^ = 0.9783); gallic acid (GAE, y = 37.817x − 31.118y, r^2^ = 0.9813); and rutin (RU, y = 10.718x + 33.928y = 10.718x + 33.928, r^2^ = 0.9748), with concentrations ranging from 0.250 to 100 µg/mL. The following concentrations were used to calculate the calibration curve for the three reference molecules: 0.25, 1, 5, 10, 50, and 100 µg/mL. The results were presented as the percentage inhibition of the DPPH radical. A 0.5% solution of each extract was prepared to evaluate the DPPH inhibition rate. The obtained DPPH inhibition values were then compared, considering the concentrations closest to those of the reference molecules.

The genomodulatory capacity was evaluated using the fluorimetric method (GEMO assay) [28] With some modifications, this is a cell-free protocol employing calf thymus double-stranded DNA (dsDNA) exposed to different concentrations of the test compounds (TC) for 30 min. Subsequently, PicoGreen^®^ (Invitrogen, Carlsbad, CA, USA), a highly sensitive fluorescent dye for dsDNA, is added, and fluorescence is emitted in proportion to the concentration of intact dsDNA. Since dsDNA in an aqueous solution undergoes spontaneous degradation leading to a decrease in fluorescence, this assay evaluated whether GuaCa extracts could reduce the degradation rate, as observed in the negative control group, which would be reflected by an increase in fluorescence levels. For this reason, the results are expressed as a percentage of the negative control.

The GEMO assay was performed in triplicate using 10 μL dsDNA samples (1 mg/mL) diluted in 5% GuaCa extract solutions. A total of 100 μL of each sample was distributed into black 96-well plates and incubated for 30 min. Subsequently, PicoGreen^®^ (diluted 1:200 in TE buffer) was added to the wells, and fluorescence was measured after five minutes at room temperature, with excitation at 480 nm and emission at 520 nm.

### 2.6. Chemical Characterization by UPLC-QToF-MS (Ultra-Performance Liquid Chromatograph Coupled to Time-of-Flight Mass Spectrometer) and Centesimal Analysis

Considering that the primary group of bioactive molecules obtained from the extracts consists of flavonoids, particularly catechins present in the chemical matrices of guarana and cocoa seed husk, a chemical characterization of a reference extract of GuaCa was conducted using (UPLC-QToF-MS, Waters Co., Milford, MA, USA). Chromatographic analyses were performed using ultra-performance liquid chromatography (UPLC) on an ACQUITY UPLC I-Class PLUS system equipped with an ACQUITY UPLC HSS T3 column, Waters Co., Milford, MA, USA (100 Å, 1.8 µm, 2.1 mm × 150 mm) at the WTS Lab of Samsung SRBR M. The mobile phase consisted of solvent A—Water + 0.1% formic acid and solvent B—Methanol/acetonitrile 25:75. Gradient elution followed a combined curve with the ratio of 0 min 95%A 5%B; 0.2 min 95%A 5%B; 10 min 5%A 95%B; 12 min 5%A 95%B; 14 min 95%A 5%B; 15 min 95%A 5%B, performed at a column oven temperature of 40 °C, with a mobile phase flow rate of 0.3 mL/min and an injection volume of 10 µL. Mass spectrometric analyses were performed using a Xevo G2 Q-TOF (Quadrupole Time-of-Flight) mass spectrometer operating in ESI- and ESI+ ionization modes. The instrument settings were as follows: capillary voltage at 2.5 kV, sampling cone voltage at 40 V, source temperature at 100 °C, and a mass range monitored from 50 to 1200 Da.

For LC-MS analysis, the samples were pre-diluted in a methanol and acetonitrile solution (25:75, *v*/*v*) at a ratio of 1:10 (*m*/*v*). This dilution phase was selected to enhance the solubilization of target compounds, ensuring proper ionization while minimizing potential interferences in analyte detection. The combination of methanol and acetonitrile was chosen due to its high miscibility and effectiveness in solubilizing both polar and semi-polar compounds, as well as its compatibility with the chromatographic system used. Following dilution, the samples were filtered through a 0.22 µm membrane to remove suspended particles, ensuring the integrity of the analytical system and the reproducibility of the results.

The relative quantification of the main components provides essential information on the chemical composition and abundance of different compounds in plant extracts or other natural sources. In practice, this process helps to understand the properties and efficacy of these products and their potential therapeutic uses, including the following: (1) Identification of Bioactive Compounds; relative quantification allows the identification of major and minor compounds in a plant extract. The major compounds are often primarily responsible for the plant’s therapeutic effects. It also serves to understand the abundance of bioactive molecules, aiding in the selection of plant extracts rich in compounds of interest for subsequent studies, such as biological activity tests; (2) Quality Control of Products; (3) Standardization of Plant Extracts; (4) Evaluation of Pharmacological Activity; (5) Research and Development of New Drugs. This enables the identification of molecules that can be used as prototypes for the development of nutraceutical drugs.

The creation of the mass spectrum (MS) library for the compounds of interest was performed using the Studio SDF software, version 3.5 (Quayside, Newcastle Upon Tyne, UK). This process involved several key steps to ensure that the library accurately represented the spectrometric profiles of each compound, facilitating subsequent identification and comparative analysis. The chemical identification of the compounds of interest was carried out using the Progenesis QI software version V2.3 (Quayside, Newcastle Upon Tyne, UK), an advanced platform for mass spectrometry data analysis, focusing on identifying and quantifying metabolites, lipids, and other biomolecules The centesimal analysis of GuaCa’s macro- and micronutrient composition was conducted by a commercial laboratory (Terra Co., Goiânia-GO, Brazil) using protocols described by the Association of Official Analytical Chemists (AOAC) [29].The percentage of moisture, macronutrients (proteins, lipids, total carbohydrates), fiber, minerals, and micronutrients were determined as follows: Na, N, Ca, Mg, S, Cu, Fe, Mn, Zn, Co, P, K, and F. Additionally, the concentration of the mixture and Total Digestible Nutrients (TDN, mg/g) was also determined.

### 2.7. In Vivo Toxicity Assessment

The potential cytotoxic effect of GuaCa at different concentrations was tested using the Red Californian earthworm (*Eisenia fetida*) as an experimental model. This simple and rapid model employs an organism commonly used in ecotoxicity studies and research involving extracts, nutritional formulations, and pharmaceuticals [21]. The protocol was similar to that described by Mastella et al. [15]. Briefly, the earthworms were commercially acquired, transferred, and acclimated to laboratory conditions and transferred to vials containing a tropical artificial soil (TAS) sterilized at 180 °C for 30 min before use. The GuaCa extract solution at different concentrations was added and homogenized into the TAS at 90% humidity. The survival of the earthworms was recorded on days 1, 3, 7, 14, 21, and 28. Three replicates were performed with three earthworms in each treatment.

### 2.8. In Vitro Cytotoxic and Neurofunctional Assessment

The commercial SH-SY5Y neuroblastoma lineage obtained from the American Type Cell Culture Collection (ATCC ^®^CRL-2266™) was using as an experimental model. The phenotypic change from typical undifferentiated cells to neuron-like cells was mechanically induced instead of being triggered by retinoic acid or other molecules, which could potentially interact with GuaCa and alter the results. The acute mechanical stress that induces cytomorphological modifications in SH-SY5Y cells is detailed in the following. Step 1: Before the experiments, aliquots of SH-SY5Y cells were thawed and cultured under standardized conditions. Step 2: The cells were transferred to sterilized Falcon tubes at a concentration of 1 × 10⁶ cells in standard culture medium. Step 3: The cells were then centrifuged for 5 min at 1500 rpm, forming a pellet at the bottom of the tube. Step 4: Using a test tube support rack, the tubes containing the cell pellet were frictioned at least 10 times, inducing mechanical impact. Step 5: Afterward, using a Pasteur pipette, the cells were resuspended and transferred to culture flasks or plates to undergo differentiation. Step 6: Within 24–48 h, the cells exhibited cytomorphological changes, adopting a neuron-like conformation.

In all protocols cells were cultured under standardized laboratory conditions (37 °C with 5% CO_2_) as previously described by [30]. Briefly, cells were maintained in Dulbecco’s Modified Eagle Medium (DMEM F12) with 10% fetal bovine serum supplemented with 1% penicillin/streptomycin. The cell suspension was placed in each well of a 96-well plate (1 × 10^5^ cells/well) with supplementation of different GuaCa extract concentrations with a logarithmic distribution (0, 0.1, 0.3, 1, 3, 10, 30, 100, and 300 µg/mL). All experiments were independently triplicated with 5–8 plate-wells of each treatment following in vitro procedures preconized by the Organisation for Economic Co-operation and Development (OECD) Guidance Document on Good in vitro Method Practices [31].

The viability analysis was performed in 24 h neuron-like cultures by MTT assay (3-[4,5-dimethylthiazol-2-yl]-2,5-diphenyltetrazolic bromide) [31]. Briefly, cultures were centrifuged at 2500× *g* and resuspended in phosphate buffer (PBS, 0.01 M; pH 7.4). MTT solution (5 mg/mL dissolved in phosphate-buffer solution, PBS) was added to a 96-well plate containing cell culture with different treatments and incubated for 2 h at 37 °C. The supernatant was removed and discarded, and the cells were resuspended in 200 µL DMSO. The absorbance was read at 560 nm, and high absorbance indicated a high concentration of viable cells [32].

One of the GuaCa concentrations that increased cell viability was utilized to conduct additional analyses of markers for neurogenic function by quantification of 8-Hydroxydeoxyguanosine (oxDNA), BDNF, extracellular NDUFS7, mitochondrial protein, β-Gal, and 8-oxDNA. Except for β-galactosidase, which was quantified using a spectrophotometric assay as described [33], the other markers were determined by immunoassays using Elabscience^®^ Biotechnology (Houston, TX, USA) kits according to the manufacturer. Calibration curves: BDNF: y = 0.2569x − 0.458, r^2^ = 0.9551; NDUFS7: y = 0.485x – 0.5177, r^2^ = 0.9779; oxDNA: y = −0.1955x + 1.3375, r^2^ = 0.9281.

### 2.9. In Vitro Immunomodulatory Effect of GuaCa on Human PD-PBMCs

The potential immunomodulatory effect was evaluated using primary human cultures from PBMCs obtained from volunteers with PD selected from patients at the Darlinda Ribeiro-FuNATI Polyclinic in Manaus-AM and Amazonas Fire Department Hospital (Amazonas Military Fire Department). The study is part of a project approved by the Research Ethics Committee of the Centro Universitário da Serra Gaúcha, Sociedade Educacional Santa Rita (CAAE 86055724.8.0000.5668) named “Effect of a Guaraná- and Cocoa Waste-Based Supplement, with and without Nanoformulation, on Oxidative-Inflammatory, Nutritional, Psychological, and Neuromotor Markers in Patients with Parkinson’s Disease”. All volunteers signed an informed consent form. Additionally, they were invited to participate in continued support for patients with neurological disorders (PAN Group), which offers psychological, nutritional, clinical, and nursing attention. A total of nine volunteers from 123 study participants were able at the start of the protocol to donate blood samples selected (males = 5, females = 6) 69.7 ± 14.11 years (45–88).

All volunteers were using levodopa as their primary medication. Regarding the stage of PD determined by the Hoehn and Yahr Scale [34], one volunteer was at stage 0, with no visible signs of the disease; three were at stage 1, with unilateral symptoms; two were at stage 2, with bilateral symptoms but without balance deficits; and the remaining participants were at stages 4 and 5, where they already exhibit severe disability and immobility. The volunteers were instructed not to consume foods high in antioxidants, caffeinated beverages, or alcoholic beverages, within the 24 h preceding blood collection.

The collection and isolation of the PBMCs were performed as described by Teixeira et al. [21]. Briefly, blood samples (20 mL) were collected by venipuncture into tubes containing ethylenediaminetetraacetic acid (EDTA) as an anticoagulant. The collected blood was transferred to tubes containing Histopaque^®^-1077 in a 2:1 ratio (blood:Histopaque^®^) for density gradient cell separation. The samples were then centrifuged at 252× *g* for 20 min. After centrifugation, the leukocyte layer was carefully collected and transferred to a new tube. The cells were washed with phosphate-buffered saline (PBS, pH 7.4) and centrifuged at 252× *g* for 10 min to remove residues. The samples were transferred to 1 mL of RPMI 1640 culture medium containing 10% fetal bovine serum and 1% penicillin/streptomycin at a final 1 × 10^5^ cells/mL concentration. The cells were distributed in 6- or 96-well plates, depending on the test, and were incubated at 37 °C, 5% CO_2_, and under controlled humidity conditions for 24 h before conducting the experiments. PBMCs of each volunteer were transferred for 96-plate wells containing culture medium without (Control) or with GuaCa supplementation. After 48 h, some inflammatory markers were evaluated in primary cell cultures obtained from each volunteer.

Due to limitations in the volume of blood samples obtained, the evaluated markers included lymphocyte proliferation assessed by the MTT assay, cytomorphological analysis via optical microscopy indicating a higher number of cells/or large cells or extensive debris due to cellular mortality, and levels of the pro-inflammatory cytokine interleukin-6 (IL-6), was quantified by ELISA immunoassay using Elabscience^®^ Biotechnology (Houston, TX, USA) kits according to the manufacturer. Calibration curve IL-6: y = 68.66x − 11.852, r^2^ = 0.9913.

### 2.10. Optical Microscopy Analysis

Considering that the supplementation of the culture medium with GuaCa could trigger an antigenic response in PBMCs, a complementary analysis was conducted to evaluate the cytomorphological conditions of the cultures stained with commercially obtained Panoptic dye according to the manufacturer’s instructions (Laborclin, Paraná, Brazil). The comparative analysis was performed by obtaining microphotographs evaluated using the free Image J software, (version 1.54p) developed by National Institute of Health. For this purpose, the images were converted to binary staining, allowing the determination of mean gray intensity (MGI). The average MGI was compared between treatments, considering that a higher number of cells and cellular debris would indicate a higher cell mortality rate and/or lymphocyte proliferation. For each obtained PBMC culture, 5–10 microphotographs were evaluated, and the results were expressed as mean ± standard deviation (SD).

### 2.11. Statistical Analysis

The results were analyzed using GraphPad Prism 9.5.1 software. Quantitative variables were generally compared using a one-way analysis of variance followed by the Tukey test. The effect on earthworms exposed to different concentrations of GuaCa was conducted using Kaplan–Meier survival curves. The comparison of biological markers in PBMC cultures at 6, 24, and 48 h was performed using repeated measures analysis of variance followed by Tyrei post hoc test. The in vitro results were presented as the mean ± standard deviation of each variable’s relative frequency (%). *p*-values < 0.05 were considered statistically significant.

## 3. Results

**Figure 1:**
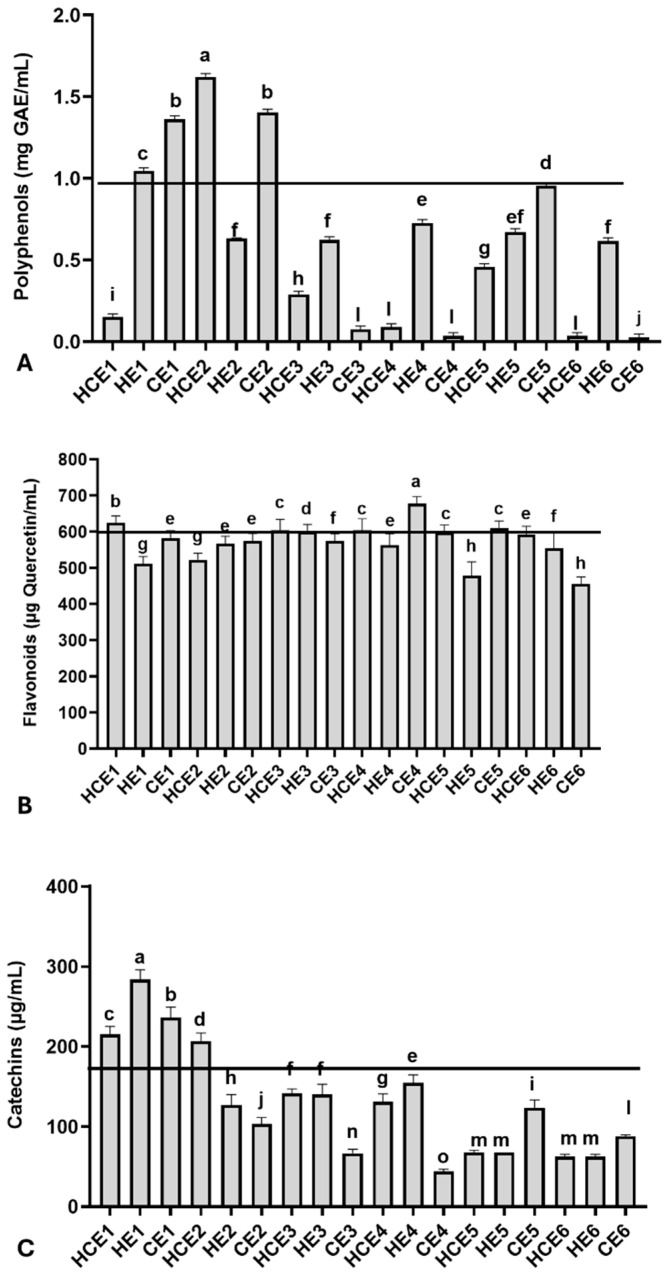
Chemical characterization of the main bioactive molecules in different combined extracts of cocoa seed husk and guarana power obtained from different temperature and pH conditions. HCE = hot and cold extraction; HE = hot extraction; CE = cold extraction; HCE = hot and cold extraction. (**A**) Total polyphenols, using gallic acid (GAE) as the reference molecule; (**B**) Total flavonoids, using quercetin as the reference molecule; (**C**) Total catechins. The values obtained from the extracts were used to estimate the 75th percentile, which served as an indicator for identifying extracts with higher concentrations of the evaluated bioactive molecule groups: total polyphenols (0.978 mg/GAE), flavonoids (572.00 µg/mL quercetin), and catechins (167.62 µg/mL). The black lines in each graph indicate levels equal to or above the 75th percentile of these chemical groups in the extracts. The GuaCa extracts were statistically compared by a one-way analysis of variance followed by a post hoc Tukey test. The comparisons made using the post hoc test were considered significantly different at *p* < 0.05. These differences were identified using different letters (a, b, c…).

Relevant total polyphenol, flavonoid, and catechin concentrations were observed among GuaCa extracts at 0.5% concentration, with some variation (Figure 1). The average polyphenol concentration was 0.601 ± 0.511 mg GAE/mL, with a minimum concentration of 0.027 mg GAE/mL observed in CE6 and a maximum concentration of 1.621 mg GAE/mL in HCE2.

Additionally, the polyphenol concentration in the dry extract was calculated. From this complementary analysis, it was observed that the HCE1 extract contained 30.02 mg GAE/g of dry extract, and the HCE2 extract had more than ten times this concentration (324.32 mg GAE/g). The 75th percentile was 0.977 mg GAE/mL for GuaCa extract, with a corresponding percentile value of 0.955 mg GAE/mL. Extracts HCE1, HE1, CE1, HCE2, and CE2 were above this threshold.

Regarding flavonoids, the average concentration in the extracts was 571.83 ± 52.22 µg quercetin/mL, with the lowest concentration (455.00 µg quercetin/mL) observed in CE6 and the highest concentration (677.33 µg quercetin/mL) found in HCE1 (Figure 1B). The following extracts had flavonoid concentrations at or above the 75th percentile, calculated at 572.00 µg quercetin/mL: CE4, HCE1, HCE3, HCE4, and CE5. The flavonoid concentrations in these extracts also estimated in mg of quercetin per gram of dry GuaCa extract were as follows: CE4 = 135.47 mg/g, HCE1 = 124.73 mg/g, HCE3 = 121.93 mg/g, CE5 = 121.93 mg/g, HCE4 = 120.87 mg/g.

The average catechin concentration was estimated at 128.92 ± 68.52 µg/mL in GuaCa extract, with a maximum concentration of 283.80 µg/mL found in HE1 and a minimum concentration of 43.83 µg/mL observed in CE4. The catechin concentration in HE1 was also estimated at 44.51 mg catechin per gram of dry GuaCa extract. The following extracts had catechin concentrations at or above the 75th percentile, calculated at 167.62 µg quercetin/mL: HCE1, HE1, CE1, and HCE2.

**Figure 2:**
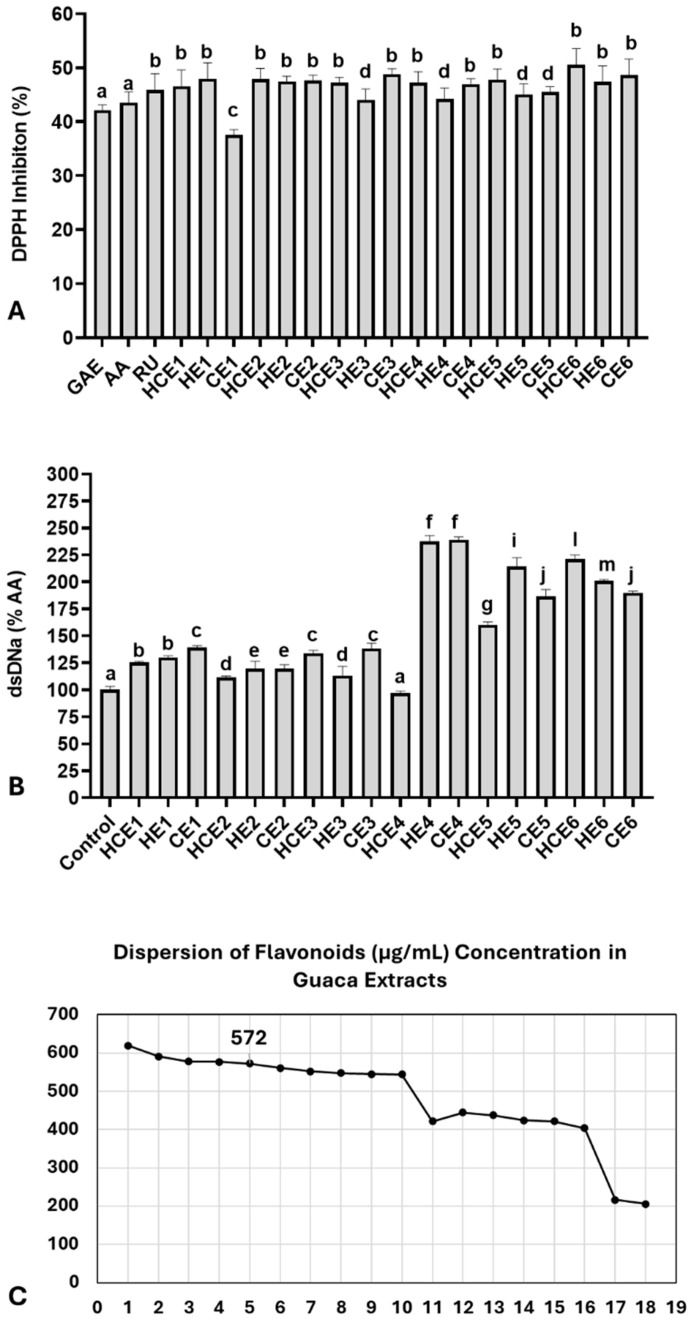
Antioxidant and genoprotective capacities of combined extracts of cocoa seed husk and guarana powder obtained under different temperature and pH conditions. HCE = hot and cold extraction; HE = hot extraction; CE = cold extraction; HCE = hot and cold extraction. (**A**) Antioxidant capacity evaluated by the DPPH assay. Data are presented as % of DPPH radical inhibition of extract solutions at 0.5% concentration, compared with three antioxidant molecules: AA = Ascorbic acid (1 µg/mL), RU = Rutin (0.25 µg/mL), and GAE = Gallic acid (1 µg/mL). These antioxidant concentrations were selected based on a reference curve, which indicated the closest DPPH inhibition range relative to the extracts (42–45%). (**B**) Genomodulatory capacity was determined by double-strand (ds) DNA concentration, quantified using fluorescent DNA PicoGreen^®^ dye. The spontaneous degradation rate of dsDNA was assessed by comparing dsDNA concentrations between the control group and the extracts. Data are presented as % of the negative control. (**C**) Dispersion graph identifying the GuaCa extract positioned closest to the 75th percentile of flavonoid concentrations (HCE3 = 572 µg/mL). Statistical comparisons for DPPH and genomodulatory (GEMO) assays were performed using a one-way analysis of variance (ANOVA) followed by Tukey’s post hoc test. The comparisons made using the post hoc test were considered significantly different at *p* < 0.05. These differences were identified using different letters (a, b, c…). In (**B**), the letter “a” indicates the control, and extracts with the same notation are considered not significantly different from it.

Next, the antioxidant capacity of the extracts was compared with three isolated bioactive molecules using the DPPH assay (Figure 2A). At a 0.5% concentration, the extracts exhibited an average DPPH inhibition rate of 46.59 ± 2.77%. This inhibition rate was slightly higher than that observed for: 1 µg/mL of ascorbic acid (AA): 43.57 ± 2.00%; 0.25 µg/mL of rutin (RU): 45.91 ± 1.50%; 1 µg/mL of gallic acid (GAE): 42.14 ± 1.50%. The DPPH inhibition rate per gram of extract was estimated to be 93.18% on average. A comparison of the antioxidant capacity among the extracts revealed the lowest inhibition rate in CE1 (37.54 ± 1.00%) and the highest inhibition rate in HCE6 (50.55 ± 1.70%). The 75th percentile of the DPPH inhibition rate was calculated at 47.89%, occurring in the following extracts: HE1, HCE2, CE6, and HCE6. However, most extracts (n = 15) exhibited a DPPH inhibition rate above 45%.

The genomodulatory capacity was also evaluated by exposing dsDNA solutions to 0.5% of each GuaCa extract (Figure 2A). On average, the extracts increased dsDNA concentrations compared to the control (159.86 ± 47.10%). The extract with the lowest dsDNA percentage was HCE4 (97.10 ± 3.1%), while the highest was CE4 (239.10 ± 4.1%). The results indicated that the extracts did not exhibit genotoxic activity, as they did not significantly reduce dsDNA concentrations compared to the negative control. In fact, only HCE4 had dsDNA concentrations similar to the control, while all other extracts showed significantly higher dsDNA concentrations.

These findings suggest a potential genoprotective effect of GuaCa extracts, considering the spontaneous degradation rate of dsDNA under experimental conditions. For this reason, additional analysis using dsDNA exposure to H_2_O_2_ was not conducted. Overall, no single extract stood out regarding polyphenol, flavonoid, or catechin concentrations. Additionally, the antioxidant capacity analysis showed relatively similar effects, indicating that the vast majority of extracts could have relevant biofunctional potential. Therefore, we selected an extract with a flavonoid concentration near the 75th percentile (Figure 2C).

To this end, the extract closest to the 75th percentile of the total flavonoid concentration was chosen, considering it relevant with a 25% margin of variation above and below the entire set of extracts. The percentile was 572.00 µg/mL; therefore, the closest identified extract was HCE3. A dispersion graph confirmed the position of its extract, shown in Figure 2C. HCE3 extract was obtained from an aqueous solution without additional acidification, with an initial temperature of 85 °C maintained for 4 h, followed by cooling and storage at 4 °C for 24 h. We also highlight that the proportion of CBS to guaraná in the HCE3 extract was 1:0.20.

The GuaCa HCE3 extract also remained within the 50th to 75th percentile for polyphenol and catechin concentrations, while also exhibiting elevated antioxidant and genoprotective capacity levels.

**Figure 3:**
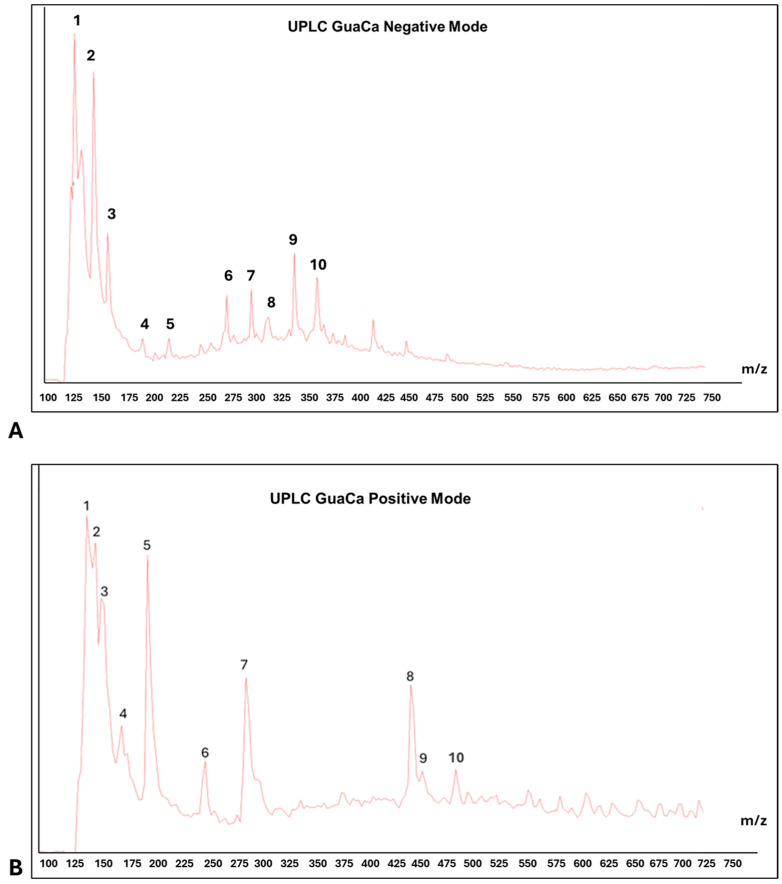
Representative chromatograms of HCE 3 GuaCa extract: (**A**) UPLC—negative mode. Main ten peaks identification: (1) Catechin, (2) Polypeptides with 4–5 Amino Acids, (3) Quercetin, (4) (-) Epicatechin, (5) (-) Epigalocatechin galate EGCG, (6) 3-O-Caffeoylquinic Acid, (7) Chlorogenic Acid, (8) Caffeoylquinic Acid, (9) Caffeic Acid Ester; (10) Chlorogenic Acid; (**B**) PLC—positive mode. Main ten peaks identification: (1) Benzolic Acid, (2) Phenylacetic Acid, (3) Alanine, (4) Caffeine, (5) Tiyrosine, (6) Quinic Acid/Chlorogenic Acid and Caffeic Acid, (7) Eicosatrienoic Acid (C20:3), (8) Elagic Acid, (9) Kampferol.; (10) Fragmented Catechin.

Centesimals analysis of the GuaCa extract revealed that it contains a significant concentration of proteins in its composition and several micronutrients that serve as cofactors in various metabolic pathways (Table 2). Chemical characterization by (UPLC-QToF-MS) identified 10 relevant peaks in both negative and positive modes in the composition of the HCE 3 extract (Table 3 and Figure 3). Among the identified molecules, four flavonoids stand out in the negative mode (catechin, quercetin, epicatechin, and EGCG), and one flavonoid in the positive mode (kaempferol). In calculating the bioactive components’ relative quantity (RQ) in the negative mode, catechin was the most abundant molecule. Using this molecule as a reference (100%), the proportion of flavonoids in the extract was estimated at 50.44%.

LC-MS also detected polypeptides, tyrosine, and alanine amino acids, consistent with the high protein content identified through centesimal analysis. In addition to these molecules, caffeine and benzoic acid were among the largest peaks detected by UPLC. In this context, as expected, the HCE 3 extract obtained from cocoa seed husk and guarana presents a composition rich in flavonoids and proteins.

**Figure 4:**
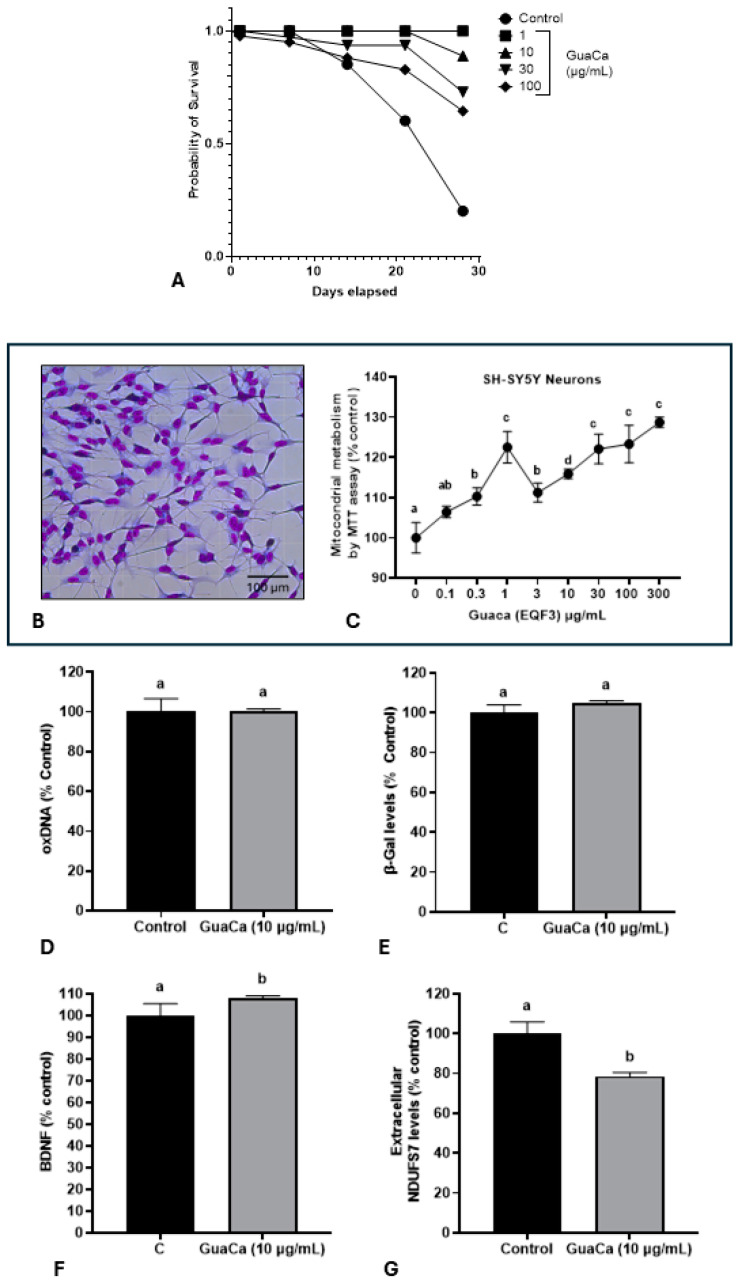
Safety and Efficacy Indicators of the GuaCa Extract (HCE3) compared with control. (**A**) Kaplan–Meier survival curves of *Eisenia fetida* worms raised in an artificial medium supplemented with different extract concentrations (µg/mL). Different letters indicate statistically significant differences in survival at *p* < 0.05. (**B**) Microphotograph showing neuron-like cells differentiated from the SH-SY5Y cell line. (**C**) Mitochondrial energy metabolism determined by the MTT assay in SH-SY5Y cultures, cultured in different concentrations of the GuaCa HCE 3 extract. Statistical comparisons were made using one-way ANOVA followed by the Tukey post hoc test. (**D**–**G**). Comparison using Student’s *t*-test of control and GuaCa-supplemented (10 µg/mL) cultures for levels of oxidized DNA determined by quantification of 8-Hydroxydeoxyguanosine (oxDNA), the cellular aging marker enzyme β-Gal, levels of the neural function marker BDNF and mitochondrial damage determined by extracellular NDUFS7 protein concentration. The data from three replicates with at least five repetitions are presented as mean ± SD. The comparisons made using the post hoc test were considered significantly different at *p* < 0.05. These differences were identified using different letters (a, b, c…). The letter “a” refers to the control group, against which all other treatments are compared.

Subsequently, it was evaluated whether the HCE 3 extract at four different concentrations could have a toxic effect on *Eisenia fetida* earthworms. The GuaCa concentrations of 1, 10, and 30 µg/mL significantly increased the lifespan compared to control worms or those raised in a medium supplemented with 100 µg/mL of the extract (Figure 4A).

The potential in vitro effect of GuaCa of differentiated SH-SY5Y neuron-like cells (Figure 4B) was evaluated. As shown in (Figure 4C), GuaCa supplementation at different concentrations (3 to 300 µg/mL) induced an increase in the energetic metabolism indicating higher cellular viability than controls. However, GuaCa did not affect oxDNA levels, a marker of DNA damage, nor β-Galactosidase levels, an indicator of cellular aging (Figure 4D,E). Although the levels of the neurogenic protein BDNF significantly increased compared to the control, these differences were marginal, suggesting more of a trend rather than a definitive result. The extracellular levels of the NDUSF7 protein, a marker of mitochondrial damage, decreased. Thus, the collective results indicate that GuaCa possesses some potentially beneficial functional properties in neuron-like cells (Figure 4F,G).

**Figure 5:**
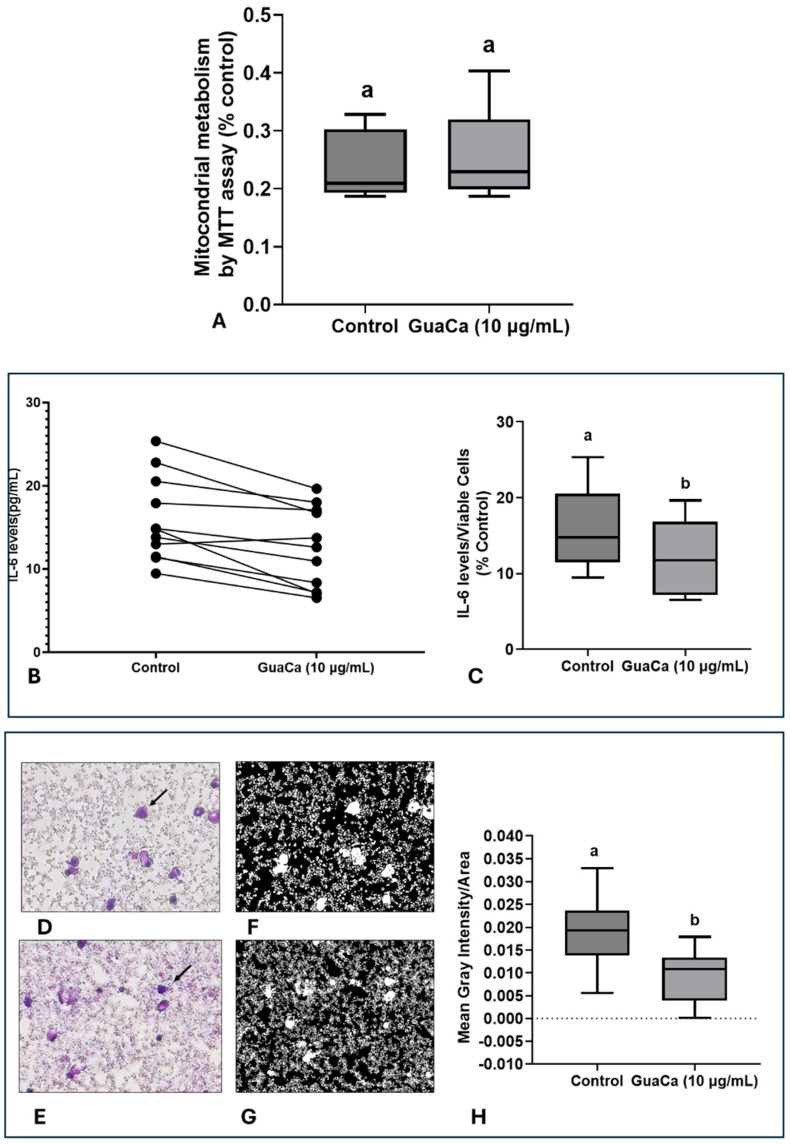
PBMCs 48-Hour Cultures Obtained from Parkinson’s Disease (PD). Volunteers with and without HCE3 GuaCa Supplementation at a Concentration of 10 µg/mL. (**A**) Energetic metabolism indicating cellular proliferation assessed by the MTT assay; (**B**) Comparison of individual IL-6 levels quantified by immunoassay in PD-PBMC cultures with and without GuaCa supplementation; (**C**) Maximum and minimum values of the pro-inflammatory cytokine IL-6; (**D**,**E**) Representative microphotographs of PD-PBMC control cultures stained with Panoptic dye. 40X magnification (**F**,**G**) cultures supplemented with GuaCa extract at 10 µg/mL. Arrows indicate mononuclear cells stained in violet. Transformation of the same microphotographs into binary staining to determine mean gray intensity, which indicates a higher quantity of cells, larger cells, or cellular debris present in the extracellular culture medium (**H**). In this case, lighter or whitish areas indicate a lower presence of cells or debris, while darker areas represent a higher concentration of cells, cell clusters, extensions, or debris. The analysis was performed using at least eight micrographs from the optical field of each treatment. These images were then transferred to ImageJ software for further analysis. Statistical comparison between cultures with and without exposure to the GuaCa extract was performed using Student’s *t*-test, and different letters indicate significant differences at *p* ≤ 0.05. The comparisons made using the post hoc test were considered significantly different at *p* < 0.05. These differences were identified using different letters (a, b). The letter “a” refers to the control group, against which all other treatments are compared.

In PBMC cultures, no significant differences were detected in MTT levels, indicating that GuaCa did not induce an acute antigenic response (Figure 5A). However, IL-6 levels were lower in PBMC cultures supplemented with GuaCa compared to controls. Figure 5B presents the results for each volunteer’s PBMC culture, comparing those with and without GuaCa supplementation, and Figure 5C shows the mean, maximum, and minimum IL-6 levels in cultures with and without GuaCa supplementation. The analysis of PBMC cultures via optical microscopy also showed a reduced number of debris and larger cells when supplemented with GuaCa (Figure 4D–G).

## 4. Discussion

This study presents a combined extract rich in flavonoids derived from cocoa seed husk and guarana powder, exhibiting neurofunctional and immunomodulatory properties that could serve as an ingredient in producing relevant nutraceuticals for patients diagnosed with PD. There is substantial evidence that bioactive components present in functional foods and dietary supplements could have beneficial effects for PD, considering the main aspects of the pathogenesis of this neurodegenerative disorder [5]. However, most studies focus on evaluating specific molecules or phytotherapeutics plants, highlighting the need to develop new specific functional products specifically targeted at PD. Within this perspective and considering the significant role of flavonoids in antioxidant function, genoprotection, mitochondrial protection, and immune modulation [5], GuaCa was delineated and formulated.

It is important to highlight aspects related to the extraction of the GuaCa extract. In general, many studies in pharmacognosy assess the functional effects of extracts obtained under a single extraction condition. While these studies are valid, an initial evaluation of variables that may influence extraction efficiency can be particularly relevant, especially when the goal is to obtain specific chemical groups. Based on published evidence, flavonoid-rich supplements may have potentially beneficial effects for neurodegenerative conditions such as PD [5]. Other bioactive molecules, such as caffeine, could also be relevant [14,35]. For this reason, the present study evaluated 18 extracts obtained under different pH and temperature conditions.

Another factor that guided the design of the GuaCa extraction conditions was the choice of solvents. In studies focusing on the isolation of bioactive compounds and functional analyses, extracts are commonly obtained using solvents, particularly alcohol. However, given that the GuaCa supplement is designed with potential industrialization in mind, we opted for aqueous extraction based on the following considerations: (i) Lower environmental impact—water is a non-toxic and biodegradable solvent, reducing chemical waste generation and the need for special disposal. The use of alcoholic solvents can lead to volatile emissions and environmental contamination risks; (ii) Reduced industrial costs—water is significantly cheaper and more accessible than ethanol or methanol, lowering production costs. It eliminates the need for distillation and solvent recovery processes, which require energy and specialized equipment. Additionally, it reduces regulatory costs associated with the handling and storage of flammable solvents; (iii) Safety and ease of handling—Using water eliminates flammability and toxicity risks, making the process safer for workers. It does not require specialized facilities for storing flammable solvents, reducing industrial safety requirements.

However, when the extracts were obtained, one notable result was that the extraction conditions did not show a linear response in the yield of bioactive molecules. For instance, the polyphenol content of HCE2 was significantly higher than that of HCE1, whereas the polyphenol content of HCE4 was significantly lower than that of HCE3. Since all extracts were produced simultaneously using the same substrates, these discrepancies do not appear to have resulted from variations in the raw material origin or experimental procedures conducted at different time points. One possible explanation for these differences may be related to the thermal sensitivity of polyphenols [22] in combination with pH interactions, which could result in a higher concentration of polyphenols in HCE2 compared to the other extracts.

The CBS was the primary source of flavonoids, as it is a residue rich in flavonoids and other chemical and nutritional components, and is produced in large quantities by the cocoa production chain [26]. However, guarana was added due to the limited number of studies specifically related to the functional properties of CBS. Guarana possesses neuroprotective properties against pollutants such as methylmercury [11], drugs like the chemotherapeutic vincristine [36], and neuropathogenic molecules such as the amyloid-beta peptide [10].

The study conducted here initially produced different extracts by combining cocoa seed husk and roasted ground guarana seed powder using water as the solvent. This choice was based on the premise that if the extracts exhibit relevant functional properties, they can later be enhanced using more efficient and non-polluting extraction techniques. Furthermore, the conception of the extract assumed that it can be used as an ingredient for the production of traditional nutraceuticals and nanoformulations that allow for oral or transdermal absorption, which is especially relevant for patients with mild to moderate dysphagia, which is common in PD and other neurodegenerative conditions.

Results from extracts showed that, despite variations in polyphenols, flavonoids, and catechin content, almost all extracts exhibited satisfactory concentrations of these natural chemicals. The bioactive components in the extract are consistent with those described in the literature [20]. The concentrations of total phenolics, flavonoids, and catechins observed in GuaCa were not higher than those found in extracts utilizing solvents such as alcohol and ethanol, which enhance extraction efficiency [7,37]. However, the formulation obtained herein focused on evaluating a solvent-free extraction process, avoiding using solvents that may be environmental pollutants or could increase processing costs if produced industrially.

In this context, the complementary evaluation of the main chemical components of the GuaCa extract via UPLC-QToF-MS is relevant to understanding their potential contribution to the observed neurofunctional and immunomodulatory effects. It is important to highlight that the positive and negative modes of UPLC-MS refer to the ionization methods used in mass spectrometry. The positive mode is preferred for compounds that tend to form positively charged ions (cations), such as amines, alkaloids, and basic compounds, while the negative mode is used for compounds that form negatively charged ions (anions), such as organic acids, polyphenols—including phenolic acids, flavonoids, and tannins [38]. However, since polyphenols can also be detected in the positive mode of UPLC-MS, we opted to analyze molecules identified in both modes.

Although it is a measure of proportionality, determining the relative quantity of the molecules with the highest peaks in the GuaCa extract suggested that a substantial portion of them are polyphenols, particularly flavonoids. In the negative mode, flavonoids accounted for 50.44% of all component molecules in the top ten UPLC peaks. Among these molecules, epigallocatechin gallate (EGCG) stands out. There is evidence that this bioactive molecule, mainly present in green tea, could have beneficial effects in delaying the neurodegeneration of the substantia nigra, regardless of the origin of PD [27]. Although quercetin (QUE) is considered a senolytic molecule, which can induce the selective apoptosis of senescent cells [39]. It is also a flavonoid found in the GuaCa extract, for which experimental studies have described protective effects against the death of dopaminergic neurons and neuron-like cells used as PD models [40].

In the positive mode, kaempferol was another flavonoid detected among the top 10 UPLC peaks. Previous studies have reported the neuroprotective effects of the flavonol kaempferol against various apoptosis- and necrosis-inducing insults associated with PD [41]. In summary, all these flavonoid molecules observed in the GuaCa extract exhibit significant antioxidant and immunomodulatory effects, as they can mitigate neuroinflammatory states present in neurodegenerative diseases such as PD.

In addition to flavonoids, LC-MS detected phenolic acids, among the highest molecular peaks, and benzoic acid, an aromatic carboxylic acid naturally used as an antibacterial and antifungal preservative in foods and feeds. Some studies suggest that appropriate benzoic acid levels might improve gut functions by regulating enzyme activity, redox status, immunity, and microbiota. However, since benzoic acid naturally occurs in plants and can also be added to prevent microbial contamination, we are uncertain whether the origin of this molecule in GuaCa is from the raw materials used in its production or if it was added to these commercially obtained products [42].

The proximate analysis showed that the GuaCa extract exhibits protein concentrations close to those reported in other studies reviewed by [8], where only cocoa bean shell was evaluated. Additionally, LC-MS analysis in the negative mode identified polypeptides with 4–5 amino acids among the ten major detected peaks, potentially including the amino acid tryptophan. Two peaks associated with key amino acids, tyrosine, and alanine, were also detected in the positive mode. Protein consumption is of great interest due to the direct and indirect effects of specific amino acids (AAs) on disease progression and their interaction with levodopa medication. Furthermore, evidence suggests that certain types of polar amino acids, such as tyrosine and alanine, which were among the significant peaks in GuaCa, may play crucial roles.

For example, tyrosine plays a crucial role in PD, as it is the direct precursor of dopamine, the neurotransmitter whose deficiency is directly associated with disease progression [43]. As highlighted in the review by Havelund et al. [44] metabolomics studies indicate that alanine levels may be altered in the plasma and cerebrospinal fluid of PD patients. Therefore, reduced alanine levels could be associated with mitochondrial dysfunction and impaired energy metabolism in PD. In this context, the presence of these amino acids in the GuaCa extract could be potentially beneficial.

The chemical and nutritional composition of GuaCa and its potential functional effects align with the findings from the experimental protocols that were conducted. Firstly, the extract exhibited low toxicity in worms exposed to various concentrations of GuaCa over 28 days. Earthworms, which are highly sensitive to toxic agents, have also been used as experimental models for PD through exposure to rotenone, which inhibits mitochondrial complex I [11]. Based on these findings, further testing in earthworms could explore GuaCa’s potential to attenuate rotenone’s neuromotor and neuroinflammatory effects. In SH-SY5Y neuron-like cultures, GuaCa also showed no visible cytotoxic effects, with cell viability increasing, suggesting a positive effect on cell cultures.

Even at a relatively low concentration of 10 µg/mL, GuaCa demonstrated relevant effects, as detailed below. Although GuaCa showed some capacity to reduce DNA degradation rates in GEMO assay, no significant reduction in oxDNA levels was observed in neuron-like cells, indicating no apparent preventive effect. However, given that PD is associated with DNA damage, it is important to assess whether GuaCa might exhibit a more effective genoprotective effect under pathological conditions.

Conversely, the significant reduction in extracellular NDUSF7 protein levels supported the observed increase in neuron-like survival rates, indicating that GuaCa may help reduce mitochondrial dysfunction associated with PD development and progression [2,3].

One of the most notable neurofunctional actions observed was borderline increased BDNF levels in cells supplemented with GuaCa. Even though this effect was not very intense, the tendency of GuaCa to modulate it still needs to be analyzed. BDNF, a member of the neurotrophin protein family, plays a direct role in neuronal survival, regulation, and memory function, mainly through interaction with the tyrosine kinase enzyme, which catalyzes ATP phosphate group transfer to tyrosine residues in target proteins. The interaction between the BDNF and TRkB enzyme enhances neurogenesis, synaptic plasticity, and neuroprotection, making it a promising therapeutic target for mental and neurodegenerative diseases like PD [13]. It is possible that higher concentrations of GuaCa could enhance its effect on BDNF production. Additionally, this effect may be relevant in experimental in vitro and in vivo models using molecules that induce alterations similar to those observed in PD. Therefore, further investigations are necessary to definitively confirm whether GuaCa modulates molecules such as BDNF.

We also investigated whether GuaCa could directly modulate the β-Gal enzyme, a known marker of cellular senescence that increases with cell aging [45]. No action of GuaCa on β-Gal modulation was observed in SH-SY5Y dopaminergic neuron-like cells. However, it is possible that in experimental models of PD, GuaCa may have some effect on β-Gal modulation.

Finally, the immunomodulatory potential of GuaCa was assessed in PBMCs from PD patients. PD is generally associated with neuroinflammatory states, leading to increased levels of pro-inflammatory cytokines such as IL-6. Most individual PBMC cultures showed reduced IL-6 levels, with cytomorphological patterns indicating decreased cell size and debris concentration.

Further studies are needed to (1) determine the absolute quantity of the main GuaCa molecules; (2) evaluate the bioavailability potential of GuaCa’s bioactive molecules using alternative delivery systems, such as sublingual solutions, gels, and transdermal creams; (3) assess the stability of the formulated product; and (4) conduct preclinical studies on GuaCa’s potential functional effects in attenuating oxidative stress, DNA damage, and neurodegenerative and neuroinflammatory states associated with PD.

Overall, the results from the protocols conducted here suggest that GuaCa may offer some preventive action against PD-related changes, such as mitochondrial damage and altered BDNF levels.

## Figures and Tables

**Table 1:** Formulae’s Aqueous Extracts, from different proportions of CBS and Guarana Powder.

Extracts *	CBS and Guarana Ratio	Initial Temperature	pH
HE1	1:0.1	85 °C	7.0
HE2	1:0.1	85 °C	5.0
HE3	1:0.2	85 °C	7.0
HE4	1:0.2	85 °C	5.0
HE5	1:0.5	85 °C	7.0
HE6	1:05	85 °C	5.0
CE1	1:0.1	4 °C	7.0
CE2	1:0.1	4 °C	5.0
CE3	1:0.2	4 °C	7.0
CE4	1:0.2	4 °C	5.0
CE5	1:0.5	4 °C	7.0
CE6	1:05	4 °C	5.0
HCE1	1:0.1	85 °C/4 °C	7.0
HCE2	1:0.1	85 °C/4 °C	5.0
HCE3	1:0.2	85 °C/4 °C	7.0
HCE4	1:0.2	85 °C/4 °C	5.0
HCE5	1:0.5	85 °C/4 °C	7.0
HCE6	1:05	85 °C/4 °C	5.0

* Hot extracts were identified with the abbreviation HE, cold extracts with the abbreviation HC, and extracts processed at both temperatures with the abbreviation HCE.

**Table 2:** Centesimal composition of GuaCa HCE 3 aqueous extract obtained from cocoa seed husk and guaraná powder.

Nutrient Specifications	Values
Total Calories (Kcal/100 g)	419.9
Carbohydrates (CHO) (g/100 g)	68.5
Protein (g/100 g)	16.5
Fat (ether extract) (g/100 g)	8.8
Mineral matter (mg/g)	6.2
Minerals (mg/g)	
Na	0.3
N	26.4
Ca	4.3
Mg	2.8
S	500
Cu	29
Fe	104
Mn	33
Zn	68
Co	4
P	4.4
K	16.8
F	10
Moisture	6.5
Total Digestible Nutrients (TDN, mg/g)	78.1
Dry Matter (mg/g)	93.5

Na: sodium; N: nitrogen; Ca: calcium; Mg: magnesium; S: sulfur; Cu: copper; Fe: iron; Mn: manganese; Zn: zinc; Co: cobalt; P: phosphorus; K: potassium; F: fluorine I.

**Table 3:** Identification of main molecules by LC-MS of HCE 3 GuaCa extract at a 1:20 Concentration.

Peak	m/z[M] ± SE (ppm)	Fragment [M]+	Abundance	Molecules	Chemical Formula	RQ (%) *
**Negative mode**						
**Flavonoids**						
1	289.09/4.07	1.31	2,129,822	Catechin	C_15_H_14_O_6_	20.48
3	591.17/2.14	3.65	1,941,788	Quercetin	C_15_H_10_O_7_	18.67
4	579.190/5	245	1,199,965	(-) Epicatechin	C_15_H_14_O_6_	11.54
5	333.17/5	152.016	1,028,357	(-) Epigalocatechin galate EGCG)	C_22_H_18_O_11_	9.89
**Other Polyphenols**						
6	364.09/0.018	162.08	933,315	3-O-Caffeoylquinic Acid	C_16_H_18_O_9_	7.48
7	191.12/5	162.08	444,048	Chlorogenic Acid	C_16_H_18_O_9_	3.55
8	324.09/5	n/a	198,074	Caffeoylquinic Acid		1.58
9	259.16/0.09	43.98	191,534	Caffeic Acid Ester	C_9_H_8_O_4_	1.53
**Polypeptides and amino acids**						
2	499.18/5	n/a	2,334,452	Polypeptides with 4–5 Amino Acids	-	22.44
**Positive Mode**						
**Other polyphenols**						
	204.15/0.010	64.01	742,057	Quinic Acid/Chlorogenic Acid	C_7_H_12_O_6_ C_16_H_18_O_9_	22.43
6	212.10/0.09	77.06	115,169	Caffeic Acid	C_9_H_8_O_4_	3.48
8	258.13/0.015	1.11	75,374	Elagic Acid	C_14_H_6_O_8_	2.28
9	344.14/0.014	193.14	742,057	Kampferol	C_15_H_10_O_6_	22.43
**Polypeptides and amino acids**						
3	132.10/0.04	44.01	366,825	Alanine	C_3_H_7_NO_2_	11.09
5	136.07/0.09	43.99	187,709	Tiyrosine	C_9_H_11_NO_3_	5.67
**Xantines**						
	195.07/−0.75	n/a	218,822	Caffeine	C_8_H_10_N_4_O_2_	6.61
**Other molecules**						
1	276.14/0.06	15.99	399,817	Benzolic Acid	C_7_H_8_O_2_	12.08
2	182.08/−0.81	n/a	382,073	Phenylacetic Acid	C_8_H_8_O_2_	11.55
7	295.16/0.015	34.11	78,926	Eicosatrienoic Acid (C20:3)	C_20_H_34_O_2_	2.39

* QR = relative quantity of each molecule calculated by abundance considering the higher peak as 100%.

## Data Availability

The original contributions presented in the study are included in the article; further inquiries can be directed to the corresponding author.
